# Food-Grade Microencapsulation Systems to Improve Protection of the Epigallocatechin Gallate

**DOI:** 10.3390/foods11131990

**Published:** 2022-07-05

**Authors:** Mathis Ralaivao, Jade Lucas, Fernando Rocha, Berta N. Estevinho

**Affiliations:** 1 LEPABE—Laboratory for Process Engineering, Environment, Biotechnology and Energy, Department of Chemical Engineering, Faculty of Engineering, University of Porto, Rua Dr. Roberto Frias, 4200-465 Porto, Portugal; mathis.ralaivao@enscm.fr (M.R.); jade.lucas@enscm.fr (J.L.); frocha@fe.up.pt (F.R.); 2 ENSCM—Ecole Nationale Supérieure de Chimie de Montpellier, 8 Rue de l’Ecole Normale, CEDEX 5, 34296 Montpellier, France; 3 ALiCE—Associate Laboratory in Chemical Engineering, Faculty of Engineering, University of Porto, Rua Dr. Roberto Frias, 4200-465 Porto, Portugal

**Keywords:** catechin, controlled release studies, epigallocatechin gallate (EGCG), microencapsulation, polyphenols, spray drying

## Abstract

Epigallocatechin gallate (EGCG) is a catechin and one of the most abundant polyphenols in green tea, and it is under research for its potential benefit to human health and for its potential to be used in disease treatments, such as for cancer. However, the effectiveness of polyphenols depends on preserving their bioactivity, stability, and bioavailability. The EGCG was microencapsulated by a spray-drying process, using different biopolymers as encapsulating agents (gum arabic, modified chitosan and sodium alginate), in order to overcome some of the limitations of this compound. The microparticles showed a diameter around 4.22 to 41.55 µm (distribution in volume) and different morphologies and surfaces, depending on the encapsulating agent used. The EGCG release was total, and it was achieved in less than 21 min for all the formulations tested. The EGCG encapsulation efficiency ranged between 78.5 and 100.0%. The release profiles were simulated and evaluated using three kinetic models: Korsmeyer–Peppas (R^2^: 0.739–0.990), Weibull (R^2^: 0.963–0.994) and Baker–Lonsdale (R^2^: 0.746–0.993). The Weibull model was the model that better adjusted to the experimental EGCG release values. This study proves the success of the EGCG microencapsulation, using the spray-drying technique, opening the possibility to insert dried EGCG microparticles in different food and nutraceutical products.

## 1. Introduction

For centuries, different civilizations used plants and their extracts in traditional medicine [1,2]. Today, catechins, and in general polyphenols, have gained interest as functional compounds in food. They are potent agents against oxidative stress; therefore, their consumption can promote the prevention of several degenerative diseases [3,4].

Catechins are found in different plants and grape seeds, and they are one of the most abundant polyphenols in green tea. They have been found to have antimicrobial, anticarcinogenic, antidiabetic, anti-oxidative, and antiviral activities [5]. The major catechins, in the green tea, include (−)-epigallocatechin gallate (EGCG), (−)-epicatechin (EC), (−)-epicatechin gallate (ECG), (−)-epigallocatechin (EGC), and their epimers. In several in vitro studies, Epigallocatechin gallate (EGCG) has been found to have the highest antioxidant activity compared to other catechins, accounting for 4.7–10.4% of the total catechins (dry weight) [5].

In recent years, Epigallocatechin gallate (EGCG) has been under research for its potential to affect human health and for its potential to be used in disease treatments, such as for cancer [6] or inflammatory diseases [7]. The important anti-cancer characteristic of EGCG has been proved in various types of cancer, and more studies are being performed [6]. EGCG has a chemo-preventive effect through inhibition of carcinogenesis process (initiation, promotion, and progression). Additionally, EGCG also has an important role in cancer management (regulating proliferation, apoptosis, angiogenesis and killing of various types of cancer cells) [6]. On the other hand, the additive or synergistic effect of EGCG with chemo preventive agents was also verified, by reducing their toxicities and enhancing the anticancerous effects [6].

For all these reasons, the incorporation of catechins in food products and nutraceuticals is so important. Adding catechins and especially EGCG to food and nutraceutical products could potentially benefit the people; however, there are some limitations related to bioavailability, stability and toxicity of the EGCG [8]. In the recent years, polyphenols have been applied in food processing, such as instant powder products, for example, gelatins and bread baking. However, catechins are highly unstable in free form, and they could be damaged due to degradation during food processing and food ingestion. The stability of the polyphenols and especially catechins in the food matrix is influenced by several factors, such as light, temperature, presence of metal ions, pH, and oxygen availability [5,6]. On the other hand, the oral bioavailability of catechins is less than 5%, and their half-life is short due to rapid systemic clearance [9]. The intestines can extend the residence time of the catechins and enable insufficient permeability. The catechins can also be damaged by other bioactives that exist in gastrointestinal fluid, namely by digestive enzymes. Finally, considering the number of hydrogen bonding donors and receptors in molecular structures, the cellular uptake of catechins was also found to be limited [10]. As a result, catechins usually have a relatively low bioavailability due to the absorption process [5,6].

The incorporation of active compounds such as catechins within controlled release systems might significantly increase their efficacy and the scopes of their application. Therefore, several approaches, including encapsulation, have emerged for their effective protection against degradation.

Various studies have been developed to improve the bioavailability of EGCG using other methodologies including the use of solid lipid nanoparticles [11,12], microemulsions [13], Chitosan-PEG-folate-Fe(III) complexes [14], chitosan-EGCG conjugates [15], Zein-based colloidal particles [16], polyphenol/protein binding, liposomes and layer-by-layer coating [17,18,19]. These techniques commonly involve complex formulation and the procedures can be time consuming [17,18,19]. In addition, it can be complicated to control the size and functionality of EGCG particles produced by some of these techniques.

Spray drying is an effective method to improve the stability of bioactive substances against oxidation and degradation, and to produce powdered products for food and pharmaceutical industry [20,21]. The powdered products produced are more convenient and safer to transport, store and process than, for example, liquid formulations [1,22]. It is the most relevant method in the food industry; few studies are reporting the application of the spray drying technique to encapsulate EGCG [23,24]. In 2010, Gomes et al. (2010), studied, using a spray drying process, the preparation of lipid/particle assemblies based on maltodextrin–gum arabic core (EGCG) as bio-carriers [9]. One year after, Fu et al. (2011) reported the production (by spray drying) of monodisperse epigallocatechin gallate (EGCG) microparticles [17]. In the same year, Peres et al. (2011) produced, by homogenization and spray-drying, gum arabic–maltodextrin particles loaded with EGCG, with a loading efficiency (EGCG) of around 96% [25]. More recently, the spray dryer was used to dry ternary aggregates dispersions and LF-HMP (lactoferrin—high methylated pectin) binary complex dispersion [26], to encapsulate soybean oil bodies in maltodextrin and chitosan-EGCG conjugates [15] and to protect (encapsulate) *Akkermansia muciniphila* in spray dried succinate-grafted alginate doped with epigallocatechin-3-gallate [27].

On the other hand, biopolymeric particles, thanks to their exceptional food compatibility and natural origin, have been widely used and studied for the encapsulation and delivery of functional compounds in nutraceutical and food products [16,28]. Carbohydrates such as chitosan, pectin, maltodextrin, gum arabic and sodium alginate can stabilize formulations and offer barrier protection and controlled release of the EGCG [18].

Accordingly, in the present work, it was explored the feasibility of the production of microparticles loaded with EGCG, by a spray drying process, using different biopolymers (modified chitosan, sodium alginate and gum arabic) with appropriated characteristics to promote protection and the release of the EGCG only in the small intestine, where it is absorbed, taking into account their possible and future integration into some food and nutraceutical products. It was also considered the ideal daily amount of EGCG uptake to avoid secondary effects, such as the risk of hepatotoxicity. To the best of our knowledge, there are no studies about the EGCG microencapsulation using these biopolymers (modified chitosan, sodium alginate and gum arabic), by a spray-drying process, as well as an extensive study of experimental release profiles and mechanisms of release in food basic conditions. Therefore, in the present study, EGCG microparticles were characterized and their release profiles evaluated. The Korsmeyer–Peppas, Weibull and Baker–Lonsdale models were adjusted to the experimental results, in order to clarify the mechanisms of the release involved.

## 2. Materials and Methods

### 2.1. Reagents and Solutions Used in the Microencapsulation Process

The core compound was (−)-epigallocatechin gallate that was bought from Sigma-Aldrich (St Louis, MO, USA).

Gum arabic, sodium alginate and modified chitosan were used as encapsulating agents for the preparation of the microparticles. Gum arabic and sodium alginate were bought from Sigma-Aldrich, USA. Water soluble chitosan was obtained from China Eastar Group (Dong Chen) Co., Ltd. (Shanghai, China).

The EGCG solutions were prepared with a concentration of 0.1% (*w*/*v*) and the encapsulating agent solutions (gum arabic, sodium alginate and modified chitosan) with a concentration of 1% (*w*/*v*). All the solutions used in the preparation of the EGCG microparticles were prepared with deionized water, at 21 °C. The formulation fed to the spray dryer was prepared adding 10 mL of the EGCG solution to each encapsulating agent solution.

### 2.2. Spray-Drying Process: Microparticles Production

The equipment used in the microparticles production (empty, and microparticles with EGCG) was a Mini Spray Dryer B-290 from BÜCHI (Flavil, Switzerland) with a 0.5 mm nozzle.

The conditions used in the spray dryer were optimized in previous studies [22,29,30]. Briefly, the solutions were fed to the equipment with a flow rate of 4 mL/min (15%), an inlet temperature of 120 °C was set, and the outlet temperature was around 56 °C. Air pressure and aspiration rate were set to 5–6 bar and 100% (36 m^3^/h).

The EGCG microparticles were produced to have a final percentage of 1% (*w*/*w*) of EGCG.

The product yield of the spray dryer was calculated (quantity of powder (EGCG microparticles) recovered, considering the quantity of raw materials (encapsulating and active agent used) for all the microparticles prepared (Equation (1)).

(1) $$\mathrm{Product}\;\mathrm{yield}\;\left(\%\right)=\frac{\mathrm{Mass}\;\mathrm{of}\;\mathrm{powder}\;\mathrm{obtained}\;\mathrm{at}\;\mathrm{the}\;\mathrm{spray}\;\mathrm{dryer}}{\mathrm{Amount}\;\mathrm{of}\;\mathrm{solid}\;\mathrm{raw}\;\mathrm{materials}\;\mathrm{used}\;\mathrm{in}\;\mathrm{the}\;\mathrm{initial}\;\mathrm{feed}\;\mathrm{solution}}\times 100$$

### 2.3. Microparticles Characterization

#### 2.3.1. Scanning Electron Microscopy (SEM)

The SEM analysis was performed using a Fei Quanta 400 FEG ESEM/EDAX Pegasus X4M equipment (Eindhoven, The Netherlands) at CEMUP (Centro de Materiais da Universidade do Porto). The SEM methodology is described in previous studies [22,29,30].

#### 2.3.2. Particle Size—Laser Granulometry Analysis

The determination of microparticles (empty and loaded with EGCG) size was made using a Coulter-LS 230 Particle Size Analyzer (Miami, FL, USA). The microparticles were characterized in terms of volume and number distribution as described in previous studies [22,29,30].

### 2.4. EGCG Controlled Release Studies

#### 2.4.1. Determination of EGCG Calibration Curve

To measure the quantity of EGCG released by the microparticles, a UV/VIS spectrophotometer (SPEC RES +, Sarspec, Porto, Portugal) was used. The analysis was made with a wavelength of 273 ± 2 nm. The EGCG calibration curve was prepared with nine standards (0.0001 to 0.0639 mg/mL), analysed in triplicate, and with a correlation coefficient of 0.9763.

The EGCG calibration curve is assumed by absorbance (A) versus concentration of EGCG (C), and represented by the equation, A = 20.477C + 0.0623.

#### 2.4.2. Encapsulation Efficiency and Loading Capacity

The microparticles containing EGCG were evaluated in terms of encapsulation efficiency (EE) (Equation (2)) and loading capacity (LC) (Equation (3)) [31].

(2) $$\mathrm{EE}\left(\%\right)=\frac{\mathrm{ECGC}\;\mathrm{amount}\;\mathrm{in}\;\mathrm{the}\;\mathrm{microparticles}}{\mathrm{EGCG}\;\mathrm{amount}\;\mathrm{used}\;\mathrm{in}\;\mathrm{the}\;\mathrm{formulation}}\times 100$$

(3) $$\mathrm{LC}\left(\%\right)=\frac{\mathrm{weight}\;\mathrm{of}\;\mathrm{EGCG}\;\mathrm{in}\;\mathrm{the}\;\mathrm{microparticles}}{\mathrm{Weight}\;\mathrm{of}\;\mathrm{microparticles}}\times 100$$

#### 2.4.3. EGCG Release Studies

The EGCG microparticles (fresh) were added on top of 3 mL of deionized water, in a quartz cuvette (CV10Q3500F, Thorlabs, Newton, NJ, USA), with stirring at 21 °C. The EGCG release profiles were created by recording the values of the absorbance at 273 ± 2 nm, in a continuous mode (intervals of 30 s).

#### 2.4.4. Kinetic Models

There are several mathematical models. In this study three different mathematical models (Korsmeyer–Peppas, Weibull and Baker–Lonsdale model) were adjusted to the EGCG experimental release profiles, using the excel tools, which allows the evaluation of the main release mechanisms involved in the release. These models were described in detail in previous studies of the authors and were selected considering the type of particles produced by spray drying (matrix type) [32,33].

Korsmeyer–Peppas model

The Equation (4) (Korsmeyer–Peppas model) considers the following variables: *Q_t_*/*Q*_∞_ is the fraction of EGCG released until time *t*, *K_K_* is the Korsmeyer constant and *n* is the release exponent [32,33].

(4) $$\frac{Q_{t}}{Q_{\infty }}=K_{K}t^{n}$$

Weibull model

The Equation (5) represents the Weibull model. This model is adequate to study the release profiles of matrix type drug delivery [34], which is normally the case of microparticles produced using a spray drying process [35].

(5) $$M_{t}=M_{\infty }\left[1-e^{-{\left(\frac{t-t_{0}}{\tau_{d}}\right)}^{\beta }}\right]$$

*M_t_* represents the dissolution/release (%) at time *t* (min), *M*_∞_ the dissolution/release (%) at infinite time, *t*_0_ the lag-time (min) of the dissolution (normally *t*_0_ = 0), *β* the shape parameter of the curve and *τ_d_* represents the time (min) when 63.2% of *M* has been dissolved/released.

Baker–Lonsdale model

This last model (Equation (6)) represents the drug release from spherical monolithic dispersions, and it was developed by Baker and Lonsdale (in 1974) [36,37]:

(6) $$f_{1}=\frac{3}{2}\left[1-{\left(1-\frac{M_{t}}{M_{\infty }}\right)}^{\frac{2}{3}}\right]-\frac{M_{t}}{M_{\infty }}=kt$$

where the release rate constant is represented by *k* and corresponds to the slope [3,38].

### 2.5. Statistical Analysis

All the analytical determinations were made in triplicate and the results were expressed with standard deviations associated to the measures. The results of statistical significance were analysed (at a level of significance *p* ≤ 0.05) by single factor analysis of variance (ANOVA), and Tukey’s test.

## 3. Results and Discussion

The main goal of this work was the production of EGCG microparticles using different biopolymers as encapsulating agents. The particles formed can be used in the food industry, directly as a food supplement/nutraceutical or being incorporated into other products like instantaneous formulations (gelatins), among others.

To accomplish this objective, three encapsulating agents were used: modified chitosan (water soluble), gum arabic and sodium alginate, and different types of particles were produced by a spray-drying technique. These biopolymers were selected considering their biocompatibility and low toxicity because they are going to be used for oral administration and that they must ensure the protection of this polyphenol along the gastrointestinal tract [28].

Several studies and methodologies were stablished in order to better describe the EGCG microparticles.

### 3.1. Preparation of EGCG Microparticles: Determination of the Product Yield of the Spray-Drying Process

As described by several authors, the product yield is one of the most significant aspects for the efficiency evaluation of spray drying process [3,39]. The properties of the solution (viscosity, the glass transition temperature of the composition or the adherence of the solution) used in the spray dryer will have a direct effect on the product yield. The size and weight of the micro/nanoparticles formed in the spray dryer process can also affect the product yield, as well as the type and scale of the equipment used (laboratorial or industrial scale) [2].

**Figure 1:**
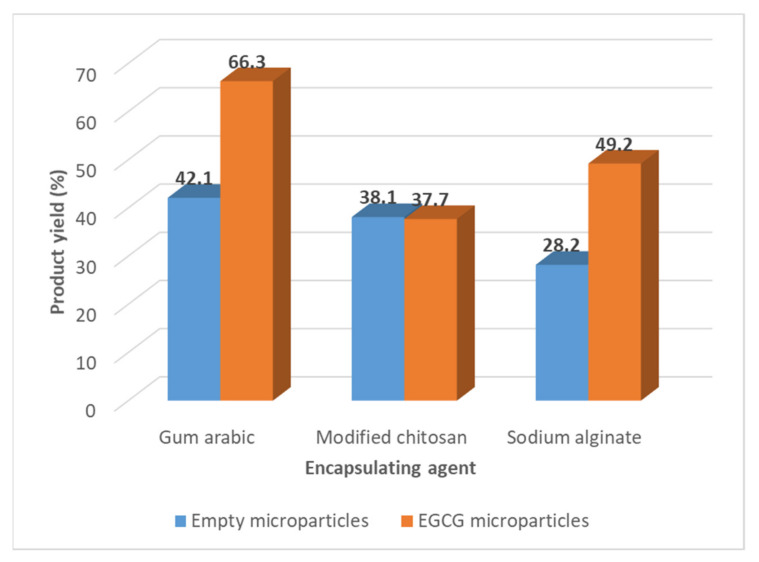
Values of the product yield of the spray drying process obtained in the preparation of different types of EGCG microparticles.

The product yield of the spray drying process, determined by the Equation (1), varied from 28.2 to 42.1% (*w*/*w*) for the empty microparticles and between 37.7 and 66.3% for the EGCG microparticles (Figure 1).

These results are in accordance with the values, described in the literature, obtained for the product yield of spray drying processes at the laboratorial scale [40,41]. The spray drying process becomes much more attractive in terms of product yield at the industrial scale, more if they have associated with the process recycling steps. In general, the incorporation of the EGCG in the microparticles increased the product yield. The gum arabic microparticles are the ones that present the highest product yield.

### 3.2. Morphology of the EGCG Microparticles: SEM

**Figure 2:**
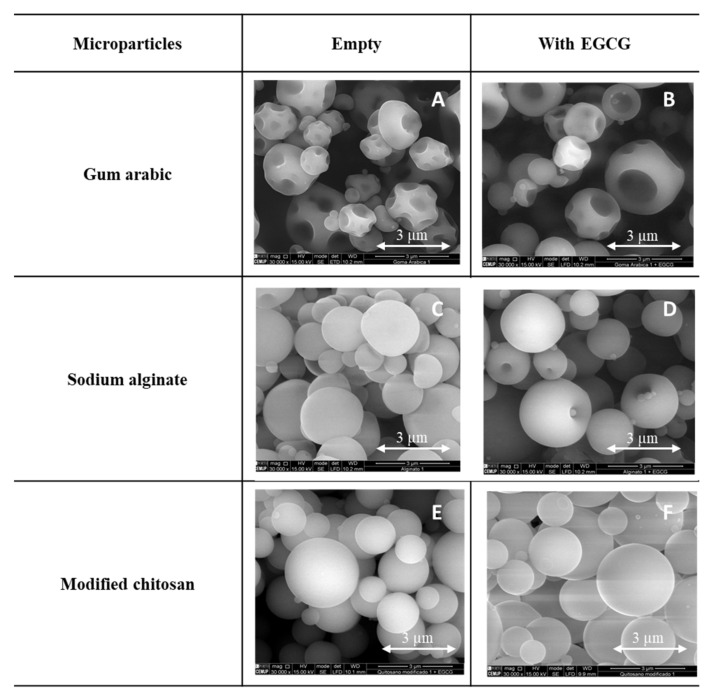
SEM images of the microparticles (empty and with EGCG), prepared with gum arabic (**A**,**B**), sodium alginate (**C**,**D**) and modified chitosan (**E**,**F**). SEM images specifications: magnification: 30,000×, beam intensity (HV): 15.00 kV, distance between the sample and the lens (WD): ±10 mm.

Different types of microparticles were prepared (empty and loaded with EGCG) with different encapsulating agents/wall materials (gum arabic, sodium alginate and modified chitosan) (Figure 2).

Different morphologies were obtained as a consequence of different factors associated with the drying process and the composition of the formulations used to produce the microparticles [42]. All the microparticles prepared presented a spherical and regular form/shape. The microparticles prepared with gum arabic (Figure 2A,B) presented several holes and wrinkles in the surface. In opposition, the particles formed with modified chitosan (Figure 2E,F) presented a very spherical and regular shape and a smooth surface. The microparticles produced with alginate (Figure 2C,D) presented some indentations on their surface. Umbilical shapes both in the empty ones and in the EGCG microparticles and rounded protuberances (small spheres) are observed. No substantial differences were detected between the microparticles loaded with EGCG and the empty ones.

Similar morphologies were also obtained and described in the literature using these encapsulating agents but with distinct core compounds: *Laurus nobilis* L. extract and gallic acid [2], Elderberry Extract and rutin [43], vitamins [33,40,44] and enzymes (β-galactosidase) [45]. From all these studies described in the literature and considering the present results, it is possible to conclude that the core material (active compound) has no substantial impact on the final morphology and shape of the microparticle. In the present work, the morphology and shape of the microparticles were more influenced by the encapsulating agent. It was also possible to observe some agglomeration and dispersion on the size of the microparticles, two characteristics associated with the spray drying process.

### 3.3. Microparticles Size: Laser Granulometry Analysis

Different factors need to be considered for the approval and commercialization of a food product, such as the particle size of the ingredients and additives of a food product. The size of the particles incorporated in a food product has a big influence on the characteristics, functionality and stability of the food products. The particles should have a dimension that allows them not to be visually or orally detected in food products [46]. Thus, the size uniformity of the particles is very important to guarantee quality, stability and similarity of the final food products [47].

In the specific case of the particles prepared by spray drying, there are some conditions of the process, which will highly influence the size of the microparticles formed, namely the nozzle type (model and diameter), the feed rate, the spray gas flow, and the solid concentration of the feed solution. For this reason, all of the operational conditions of the spray dryer were kept constant during the assays.

**Figure 3:**
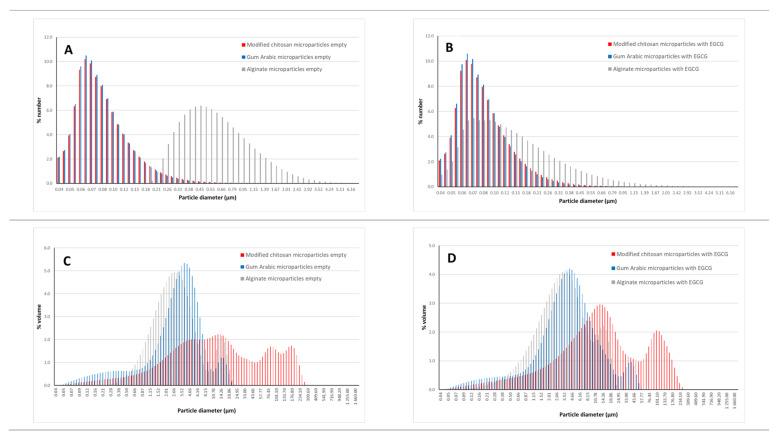
Size distribution considering a number distribution (**A**,**B**) and volume distribution (**C**,**D**) of the empty microparticles (**A**–**C**) and EGCG microparticles (**B**–**D**).

The particles were analyzed considering a differential volume and number size distribution (Table 1), and the respective size distribution graphs are presented in Figure 3.

Considering a differential number distribution, the microparticles presented a mean size around 110 nm, except the microparticles were prepared with sodium alginate. The alginate microparticles loaded with EGCG had a mean size around 210 nm and the empty microparticles of sodium alginate of 710 nm. In terms of differential volume distribution, the mean sizes are higher (ranging between 4.22 and 41.55 μm). The empty microparticles prepared with modified chitosan presented the highest value for the mean size (41.55 μm) followed by the EGCG particles prepared with modified chitosan (36.12 μm). The particles prepared with sodium alginate (4.31 (empty)—5.09 μm (EGCG)) and gum arabic (4.22 (empty)—7.22 μm (EGCG)) had smaller mean sizes.

For each type of microparticle, the difference between the mean size obtained considering a number and a volume distribution can be explained by the presence of some agglomeration. It is possible to observe in Figure 3, namely, in the figures including the volume distribution (Figure 3C,D), the existence of a bimodal/multimodal distribution, associated with the agglomeration phenomenon.

### 3.4. EGCG Release Profiles and Controlled Release Studies

**Figure 4:**
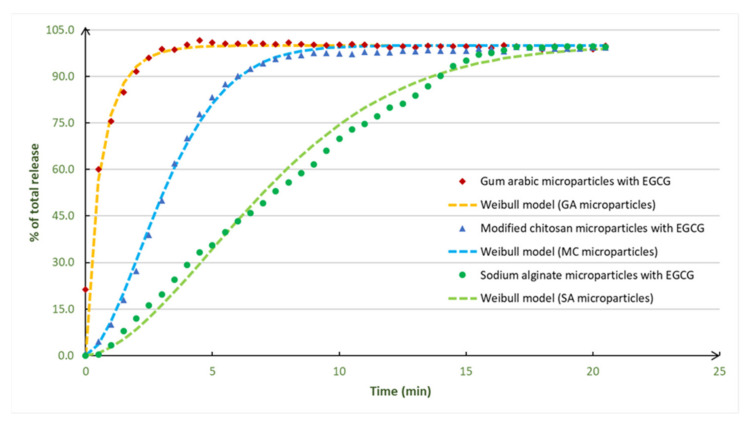
Experimental data and Weibull model adjustments for the EGCG release profiles obtained with microparticles prepared with different encapsulating agents. The experimental release profiles were obtained, in triplicate, and the variation coefficients were determined for each release time and were less than 10% for the all the release times.

The EGCG release profiles were prepared in assays with deionized water as release medium, at 21 °C (Figure 4). The deionized water (pH 5.6) was used because it can represent the most popular and simplest solvent used in food industry and in the formulation of food and nutraceutical products.

The release profiles presented two distinct zones: the first one is the release zone (high release rate), and the second (final) zone corresponds to the stabilization zone, where all the EGCG was released from the microparticles. The EGCG microparticles had a total EGCG release. The EGCG release was fast, and the fastest release was observed for the EGCG microparticles prepared with gum arabic (4 min).

Other relevant information (Table 2) is the encapsulation efficiency and the loading capacity. The encapsulation efficiency is higher, close to 100%, for the EGCG microparticles made with modified chitosan and sodium alginate, having these microparticles respectively 0.99% (*w*/*w*) and 1% (*w*/*w*) of EGCG.

Studies described in the literature reported efficiencies of encapsulation lower than the ones achieved in the present study. Ding et al. (2019) prepared by spray drying soybean oil bodies encapsulated in maltodextrin and chitosan-EGCG conjugates with the highest encapsulation efficiency of 96.68%. Peres et al. (2011) microencapsulated EGCG with a loading efficiency of 96 ± 3%.

The EGCG experimental release profiles were adjusted to three mathematical models: Korsmeyer–Peppas, Baker–Lonsdale and Weibull models. From Table 3, it is possible to observe that the model that shows the better adjustment to the experimental EGCG release profiles is the Weibull model, which was also represented in Figure 4. The EGCG microparticles prepared with gum arabic adjust well to all the three models in special to the Baker–Lonsdale model (0.993). For the EGCG microparticles prepared with modified chitosan and sodium alginate, the best model is the Weibull model.

Therefore, relative to the Weibull model, the coefficients of correlation were higher than 0.963 (value that corresponds to the EGCG microparticles prepared with gum arabic). The highest value was for the microparticles prepared with modified chitosan (0.994). According to the literature, the Weibull model is the most adequate model to evaluate the release profiles of matrix-type microparticles, typically produced by spray drying processes, which is the case of the EGCG particles prepared in the present work. Normally, the mechanisms involved on the release from these kind of particles are controlled by solvents action and by diffusion [3].

The values of the parameter *τ_d_* obtained directly from the experimental EGCG release profiles are very similar to the parameter *τ_d_* obtained from the model for all the types of EGCG microparticles. In relation to the parameter *β*, it was less than 1 (0.9) for the EGCG microparticles prepared with gum arabic and higher than 1 for the microparticles prepared with modified chitosan and sodium alginate. Thus, according to the value of the *β* (shape parameter of the curve), different conclusions can be obtained: if *β* = 1, the shape of the curve corresponds exactly to an exponential profile; if *β* > 1, the shape of the curve becomes sigmoidal with a turning point; and if *β* < 1, the shape of the curve would show a steeper increase than the one with *β* = 1.

The other model explored was the Korsmeyer–Peppas model, which was adjusted to the experimental EGCG release profiles, with correlation coefficients varying from 0.739 (EGCG microparticles prepared with sodium alginate) to 0.990 (EGCG microparticles prepared with gum arabic). The Korsmeyer–Peppas model provides information about the major mechanism responsible for the controlled release, based on the values of the “*n*” parameter. Therefore, if *n* < 0.43, the drug transport mechanism is by a Fickian diffusion, which was the case of the EGCG microparticles prepared with gum arabic. If 0.43 < *n* < 0.85, the drug transport mechanism is by an anomalous transport that happens involving both Fickian diffusion and polymer chain relaxation mechanisms, and this was the case of the EGCG microparticles made with modified chitosan. If *n* > 0.85, which was the case of EGCG microparticles prepared with sodium alginate, the active compound transport mechanism is a super case-II transport (polymer chain relaxation mechanisms). If *n* = 0.85, the active compound transport mechanism is by a case-II transport [20].

The Baker–Lonsdale model was also applied. This model clarifies the controlled release kinetics of spherical monolithic dispersions, which is the case of the microparticles prepared by spray drying, and presents coefficients of correlation ranging from 0.746 (EGCG microparticles prepared with modified chitosan) to 0.993 (EGCG microparticles prepared with gum arabic).

### 3.5. Food Application and Safety Evaluation

This work demonstrates that it is feasible to encapsulate EGCG through a spray-drying process. The biopolymers used as encapsulating agents were selected in order to release the active compound in the intestine, where the EGCG is absorbed. Microparticles containing EGCG presented high quality, spherical and regular shape and had a high microencapsulation efficiency, exhibiting a fast release that can be used in the development of new food/nutraceutical products.

However, an important question should be answered, related to how much EGCG can be added in these food products. According to the literature, green tea catechins, in particular the EGCG, present several health advantages (health-promoting effects on cancer, cardiovascular diseases, neurodegenerative diseases, and strengthening the immune system, among others) [19]. They are normally absorbed in the small intestine and bio-transformed in the liver and in enterocytes in the small intestine. However, there is the risk of hepatotoxicity for high concentrations of catechin [48]. A controlled release system is also useful in order to reduce the toxicity of the compounds. Therefore, it is important to analyze some data. The average daily intake of EGCG resulting from the consumption of green tea infusions varies from 90 to 300 mg/day [11,48]. One adult (high-level consumers), in the European Union, can achieve values up to 866 mg EGCG/day. On other hand, food supplements, for adult population, can provide a daily dose of EGCG in the range of 5 to 1000 mg/day. On the other hand, the Scientific EFSA Panel on Food Additives and Nutrient Sources added to Food (ANS) decided that there is proof from clinical experiments that ingestion of doses equal or above 800 mg EGCG/day taken as a food supplement has been shown to induce an increase in serum transaminases in treated subjects compared to control [48].

Thus, considering these values and assuming 800 mg EGCG/day as limit, it was estimated (Table 4) the amount of powder (microparticles) prepared with each of the formulations studied that should be taken as a food supplement or additive per day.

The microparticles prepared with sodium alginate were the formulation that requested the smallest amounts of product to be taken in order to comply with the intake doses associated to the EGCG. Therefore, depending on the pretended supplementation, one determined amount should be considered. As mentioned before, the microencapsulation can protect the EGCG, increasing its stability and creating the possibility to be used in a more efficient way in food supplementation.

## 4. Conclusions

Different types of microparticles were prepared (empty and loaded with EGCG) with different biopolymers as encapsulating agents (sodium alginate, gum arabic, and modified chitosan). EGCG microparticles prepared with gum arabic presented several holes and wrinkles in the surface. In opposition, the EGCG particles formed with modified chitosan presented a very spherical and regular form with smooth surface. In terms of differential volume distribution, the mean sizes ranged between 4.22 and 41.55 μm, and some agglomeration phenomena could be detected. The spray drying process was evaluated in terms of product yield of the process, which varied from 28.2 to 66.3%. The encapsulation efficiency was high, close to 100% for the EGCG microparticles prepared with modified chitosan and sodium alginate, having these microparticles respectively at 0.99% (*w*/*w*) and 1% (*w*/*w*) of EGCG. The EGCG release was totaled, and it was achieved in less than 21 min for all the formulations tested. The model that shows the better adjustment to the experimental EGCG release profiles was the Weibull model. The main mechanisms associated to the release were Fickian diffusion, Anomalous transport and Super case-II transport, for the EGCG microparticles prepared with gum arabic, modified chitosan and sodium alginate, respectively. The encapsulated formulations have advantages to reduce toxic concentrations of EGCG in the human body, which can happen for EGCG doses higher than 800 mg/day. The microparticles prepared with sodium alginate were the formulation that incorporates more EGCG and consequently requests smaller amounts of product to achieve a determined EGCG dose. Therefore, EGCG microparticles with good quality, spherical and with a regular shape were prepared with a high encapsulation efficiency and presenting a fast release. These microparticles can be used, in the future, for the incorporation into food and nutraceutical products.

## Figures and Tables

**Table 1:** Microparticles’ (empty and containing EGCG) mean size results obtained, considering a differential volume and number distribution, prepared with different encapsulating agents.

Microparticles	Mean Size (µm)	
	Differential Number	Differential Volume
Gum arabic	0.10 ± 0.00	4.22 ± 0.02
Gum arabic—EGCG	0.10 ± 0.00	7.22 ± 0.03
Modified chitosan	0.11 ± 0.00	41.55 ± 9.31
Modified chitosan—EGCG	0.11 ± 0.00	36.12 ± 5.73
Sodium alginate	0.71 ± 0.00	4.31 ± 0.06
Sodium alginate—EGCG	0.21 ± 0.10	5.09 ± 0.10

**Table 2:** Encapsulation efficiency, loading capacity and time parameters of the EGCG release.

Biopolymer Used	Encapsulation Efficiency (%)	Loading Capacity (%) (*w*/*w*)	Total Release Time (min)
Gum arabic	78.5 ± 5.5	0.79 ± 0.06	4 ± 1
Sodium alginate	100.0 ± 1.0	1.00 ± 0.01	21 ± 1
Modified chitosan	99.3 ± 1.0	0.99 ± 0.01	17 ± 1

**Table 3:** Parameters and correlation coefficients of the Korsmeyer–Peppas, Weibull and Baker–Lonsdale models applied to the experimental EGCG release profiles.

Microparticles with EGCG	Korsmeyer–Peppas Model					Weibull Model				Baker–Lonsdale Model	
	Time of the Adjust (min)	*K_K_* (min-n)	*n*	Main Mechanism Associated to the Release	R^2^	*τd_experimental_* (min)	*τd_calculated_* (min)	*β*	R^2^	*k*	R^2^
Gum arabic	2.0	0.744	0.18	Fickian diffusion	0.990	1.0	0.6	0.9	0.963	0.155	0.993
Modified chitosan	4.0	0.178	0.52	Anomalous transport	0.864	4.0	3.6	1.7	0.994	0.027	0.746
Sodium alginate	9.0	0.045	1.09	Super case-II transport	0.739	9.0	8.3	1.7	0.973	0.011	0.815

*K_K_* is the Korsmeyer constant and *n* is the release exponent. *β* the shape parameter of the curve and *τd* represents the time (min) when 63.2% of M has been dissolved/released. *k* represents the release rate constant.

**Table 4:** Estimate of the amounts of EGCG microparticles corresponding to normal intake doses of EGCG.

Doses of EGCG (mg/Day)		EGCG Microparticles (g/Day)		
		Gum Arabic	Sodium Alginate	Modified Chitosan
Normal doses—consumption of green tea infusions	90–300	11.4–38.1	9.0–30.0	9.1–30.2
Food supplements	5–1000	0.6–126.9	0.5–100.0	0.5–100.7
“Safety” dose	<800	<101.5	<80	<80.6

## Data Availability

Data that support the findings of this study are available on request to the authors.
